# Metabolic Profiling Identified a Novel Biomarker Panel for Metabolic Syndrome-Positive Hepatocellular Cancer

**DOI:** 10.3389/fendo.2021.816748

**Published:** 2022-01-26

**Authors:** Lin-Lin Cao, Yi Han, Yuanxiao Wang, Lin Pei, Zhihong Yue, Li Qin, Boyu Liu, Jingwen Cui, Mei Jia, Hui Wang

**Affiliations:** ^1^ Department of Clinical Laboratory, Peking University People’s Hospital, Beijing, China; ^2^ Department of Pharmacy, Peking University People’s Hospital, Beijing, China; ^3^ SCIEX Analytical Instrument Trading Co., Shanghai, China

**Keywords:** hepatocellular cancer, metabolic syndrome, metabolomics, L-glutamic acid, pipecolic acid

## Abstract

Metabolic syndrome (MetS) is an independent risk factor for hepatocellular cancer (HCC). Currently, there is no highly sensitive and specific biomarkers for HCC surveillance in MetS population. Metabolomics has been reported as a powerful technology for biomarker discovery. In the present study, we aimed to explore novel biomarkers with high sensitivity and specificity for MetS-positive [MetS(+)] HCC by metabolomic analysis. At first, many serum metabolites were found dysregulated in MetS(+) HCC individuals. Validation of the dysregulated metabolites by targeted metabolite analyses revealed that serum L-glutamic acid (L-glu), pipecolic acid (PA) and 7-methylguanine (7-mG) were increased in MetS(+) HCC compared to MetS group. Then a biomarker panel including L-glu, PA and alpha-fetoprotein (AFP) was identified as a novel biomarker for the diagnosis of MetS(+) HCC. Receiver operating characteristic (ROC) curve was drawn and the area under the ROC curve (AUC) was 0.87 for discriminating MetS(+) HCC from MetS group. The biomarker panel was capable of detecting small (AUC = 0.82) and early-stage (AUC = 0.78) tumors as well. Moreover, it exhibited great diagnostic performance (AUC = 0.93) for discriminating MetS(+) HCC from other MetS-associated cancers, including colorectal cancer and gastric cancer. Collectively, our study establishes a novel diagnostic tool for MetS(+) HCC.

## Introduction

Hepatocellular cancer (HCC) is the most common primary liver cancer and represents a seriously threat to human health. It is estimated to be the fourth-most frequent cause of cancer mortality in the world (1, 2). Although considerable progress has been achieved in the diagnosis and treatment of HCC during the past few decades, the prognosis of HCC is still very poor, possibly due to the lack of obvious symptoms in the early stages and the delay in diagnosis of the disease (2). It has been reported that tumors diagnosed at early stages are suitable for curative therapy, with a median overall survival (OS) of exceeding 60 months, whereas the median OS of patients with advanced-stage HCC is only 11 months (3). Therefore, early detection of HCC in high-risk populations is essential to improve the prognosis of HCC patients.

It has been widely known that the hepatitis B virus (HBV) and hepatitis C virus (HCV) are the most important risk factors for HCC at present. However, their importance is gradually decreasing, which is due to the vaccination of newborns and the effective treatment of both HBV and HCV infections (4). Increasing evidence shows that metabolic syndrome (MetS) is also a significant risk factor for HCC, regardless of other risk factors (5–7). MetS is a cluster of metabolic abnormalities including insulin resistance, dyslipidemia, hypertension and central obesity. It has been demonstrated that MetS is associated with a 1.81-fold increased risk of HCC (8), indicating the necessity of monitoring the MetS population to ameliorate HCC risk. However, there is no specific biomarker for early detection of HCC in MetS patients at present. Serum alpha-fetoprotein (AFP) is the most widely used biomarker for HCC diagnosis, but its diagnostic accuracy is not satisfactory (2). Some other circulating biomarkers, such as microRNAs (9), specific proteins (10, 11) and differentially DNA methylation (12, 13), have been identified as potential biomarkers for HCC diagnosis, but they are not specific for MetS-positive [MetS(+)] HCC and not suitable for HCC surveillance in MetS population. Therefore, it is critical to explore novel biomarkers for MetS(+) HCC.

Metabolomics, which serves as a powerful platform focusing on the comprehensive profiling of small metabolites, has provided a promising technology for biomarker discovery (14). The liver is one of the most important metabolic centers of humans, and regulates many important metabolic processes. Therefore, there is no doubt that HCC occurrence is accompanied by changes in the levels of numerous metabolites, and metabolomic analysis is particularly useful for HCC diagnosis by determining dysregulated metabolites (15). A lot of effort has been devoted to the metabolomic study on HCC using various specimens, including liver tissue, serum and urine, and many metabolites have been reported as biomarkers for HCC diagnosis and prognosis (16–21). However, most studies have focused on HBV- and HCV-associated HCC, and there is still a lack of research on the metabolic biomarkers of MetS(+) HCC.

In this study, we aimed to explore the dysregulated metabolites in MetS(+) HCC compared to MetS patients, and find potential biomarkers for HCC surveillance in MetS population. Serum metabolite profiles in MetS patients and MetS(+) HCC patients were determined by untargeted metabolomic analysis. The metabolites that were differentially expressed in MetS(+) HCC compared with MetS patients were validated by targeted metabolite analyses. In addition, diagnostic values of these biomarkers and their correlation with clinicopathologic variables of patients were also evaluated. Overall, our study explored candidate metabolite biomarkers for the diagnosis of MetS(+) HCC.

## Materials and Methods

### Study Population

In the present study, a total of 407 participants, including patients with HCC, colorectal cancer (CRC), gastric cancer (GC), MetS, and healthy controls (HC) were recruited in Peking University People’s Hospital. In the discovery stage, 32 MetS patients and 43 MetS(+) HCC patients were included and subjected to metabolomic analyses. In the validation stage, 94 HCs, 100 MetS patients, 66 MetS(+) HCC patients, 42 MetS(+) CRC patients and 30 MetS(+) GC patients were included and subjected to targeted metabolite analyses. This study was approved by the Ethics Committee of Peking University People’s Hospital and complied with the principles of the Declaration of Helsinki. Informed consents were obtained from all recruited participants.

The presence of MetS was defined as three or more of the following metabolic situations (22): (1) Central obesity: waist circumstance ≥ 90 cm in men or ≥ 85cm in women; (2) Hyperglycemia: fasting glucose (FG) ≥ 6.1 mmol/L or 2-h glucose in oral glucose tolerance test (OGTT) ≥ 7.8 mmol/L and/or confirmed diabetes that is under treatment; (3) Hypertension: blood pressure ≥ 130/85 mmHg and/or confirmed hypertension with antihypertensive therapy; (4) Fasting triglycerides (TG) ≥ 1.70 mmol/L; (5) Fasting high-density lipoprotein cholesterol (HDL-C) < 1.04 mmol/L. The diagnosis of patients with HCC, CRC and GC were confirmed by histopathology, and patients with other types of malignancy were excluded. In addition, all patients with HCC, CRC and GC included in this study had MetS. The enrolled HC subjects were healthy people who had received physical examinations. Peripheral blood samples were collected under fasting conditions before surgery. The demographic and clinical characteristics were collected from medical records and summarized in **Table 1**.

**Table 1:** The demographic and clinical variables of individuals included in this study.

	Discovery Cohort		Validation Cohort				
**Variables**	MetS	MetS(+) HCC	HC	MetS	MetS(+) HCC	MetS(+) CRC	MetS(+) GC
	N=32	N=43	N=94	N=100	N=66	N=42	N=30
**Age**	56.47 ± 11.37	59.09 ± 11.76	43.56 ± 15.02	54.39 ± 12.04	58.59 ± 9.94	66.31 ± 10.43	69.23 ± 11.36
**Gender Male/Female**	20/12	36/7	32/62	71/29	53/13	29/13	20/10
**AFP >7/≤7 ng/ml**	4/28	27/16	2/92	10/90	41/25	2/40	7/23
**FG (mmol/L)**	7.43 ± 1.43	6.91 ± 2.28	4.99 ± 0.42	8.60 ± 2.37	6.94 ± 2.70	5.46 ± 1.63	6.64 ± 3.51
**TG (mmol/L)**	1.77 ± 0.61	1.38 ± 0.61	1.03 ± 0.30	2.85 ± 1.34	1.45 ± 0.71	1.63 ± 0.51	1.56 ± 0.53
**HDL-C (mmol/L)**	0.99 ± 0.18	0.92 ± 0.25	1.29 ± 0.15	0.89 ± 0.08	0.94 ± 0.24	0.92 ± 0.18	0.96 ± 0.23
**Central obesity +/-**	16/16	22/21	10/84	44/56	40/26	31/11	22/8
**Hypertension +/-**	20/12	32/11	8/86	75/25	44/22	31/11	16/14

### Chemicals and Reagents

All solvents used in this study were of high-performance liquid chromatography (HPLC) grade. Acetonitrile was purchased from Fisher Chemical. Methanol, ammonia hydroxide and formic acid were purchased from Sigma-Aldrich. Distilled water was purchased from Watsons. Ammonium acetate was purchased from Aladdin. The isotopically-labelled internal standard mixture used in metabolomic analyses was from Biotree Biomedical Technology. L-glutamic acid (L-glu), citrulline (Citru), pipecolic acid (PA), 7-methylguanine (7-mG) and L-glu-2,3,3,4,4-d5 were purchased from Sigma-Aldrich as well.

### Measurement of Clinical Indicators

The peripheral blood sample was collected and serum was separated by centrifuging at 4000 rpm for 5 minutes for each individual. Serum levels of FG, TG, HDL-C were measured by AU5832 automatic biochemical analyzer (Beckman Coulter). Serum HBV surface antigen (HBsAg) was detected by the automatic chemiluminescent microparticle immunoassay analyzer ARCHITECT i2000 SR (Abbott Laboratories). The concentration of serum AFP was determined by an electrochemiluminescence immunoassay in Cobas e801 system (Roche Diagnostics). All measurements were performed with original manufacturers’ reagents according to the manufacturers’ instructions.

### Metabolomic Analyses

The pretreatment of serum samples was as follows. 100 μL of serum sample was mixed with 400 μL of extract solution (acetonitrile: methanol = 1: 1, containing isotopically-labelled internal standard mixture) by vortexing for 30 seconds. Then the sample was sonicated for 10 min in ice-water bath, and incubated for 1 hour at -40°C to precipitate proteins. Subsequently, the sample was centrifuged at 12000 rpm for 15 min at 4°C. The supernatant was transferred into a fresh glass vial for metabolomic analysis. The quality control (QC) sample was prepared by mixing the supernatants from all of the samples in equal amounts.

Metabolomic analyses were performed using a UPLC system (Vanquish, Thermo Fisher Scientific) coupled to a Q Exactive HFX mass spectrometer (Orbitrap MS, Thermo Fisher Scientific). An ACQUITY UPLC BEH Amide column (2.1 mm × 100 mm, 1.7 μm, Waters) was used for analysis. The mobile phase A consisted of 25 mmol/L ammonium acetate and 25 mmol/L ammonia hydroxide in water (pH = 9.75), and the mobile phase B was 100% acetonitrile. The auto-sampler temperature was maintained at 4°C, and the column temperature was set at 30°C. The injection volume was set at 2 μL, and the flow rate was set at 0.5 mL/min. The following elution gradient was applied: 0-0.5 min, 95% B; 0.5-7 min, 95%-65% B; 7-8 min, 65%-40% B; 8-9 min, 40% B; 9-9.1 min, 40%-95% B; 9.1-12 min, 95% B. The ion spray voltage was set at 3600 V in the ESI+ mode and −3200 V in the ESI− mode. The capillary temperature was set at 350°C. The sheath gas flow rate was set at 30 arbitrary units and the aux gas flow rate was set at 25 arbitrary units. The mass scan range was set from 70 to 1050 m/z. The full MS resolution was set at 120000. A stepped normalized collisional energy (10, 30, 60 eV) approach was applied for effective fragmentation. The acquisition software Xcalibur (Thermo Fisher Scientific) was used to acquire MS/MS spectra on information-dependent acquisition (IDA) mode.

### Targeted Metabolite Analyses

Calibration standard mixtures and QC samples were prepared according to the following procedures. At first, twenty serum samples were mixed in equal amounts. Then seven standard mixtures of L-glu, Citru, PA and 7-mG were prepared by 10-fold serial dilutions using the mixed serum to eliminate the matrix effect. The concentrations of these standard mixtures were determined with reference to the serum concentration ranges of these metabolites described in previous studies (23–26). QC samples (low-level and high-level) were prepared by spiking appropriate concentrations of L-glu, Citru, PA and 7-mG into the mixed serum.

Subsequently, the pretreatment of calibration standard mixtures, QC samples and serum samples was performed as follows. 100 μL of serum sample was mixed with 200 μL of extract solution (acetonitrile: methanol = 1: 1, containing isotopically-labelled internal standard L-glu-2,3,3,4,4-d5) by vortexing for 60 seconds, and centrifuged at 12000 rpm for 15 min at 4°C. Then 200 μL of the supernatant was diluted by adding 800 μL of water, and the diluted sample could be directly used for targeted metabolite analysis.

Targeted metabolite analyses were performed using a Jasper HPLC system coupled to a Triple Quad 4500MD (SCIEX). A Kinetex F5 column (3 mm × 100 mm, 2.6 μm, Phenomenex) was used for analysis. The mobile phase A consisted of 10 mmol/L ammonium acetate and 0.05% formic acid in water, and the mobile phase B consisted of 5 mmol/L ammonium acetate and 0.05% formic acid in 90% acetonitrile. The auto-sampler temperature was maintained at 4°C, and the column temperature was set at 40°C. The injection volume was set at 5 μL, and the flow rate was set at 0.4 mL/min. The following elution gradient was applied: 0-1 min, 6% B; 1-2 min, 6%-98% B; 2-3.5 min, 98% B; 3.5-3.6 min, 98%-6% B; 3.6-5 min, 6% B. The MS detection was carried out with a Turbo Spray probe in positive ion mode. The metabolites were tuned individually to get optimal signals, and multiple reaction monitoring (MRM) mode was used to monitor the specific metabolite transitions. The MRM settings are shown in **Supplementary Table 1**. The ion spray voltage was set at 5500 V, and the capillary temperature was set at 400°C. The curtain gas flow rate was set at 25 arbitrary units and the collision gas flow rate was set at 9 arbitrary units. The acquisition software Analyst MD 1.6.3 (SCIEX) was used to acquire MS/MS spectra, and MultiQuant MD 3.0.3 (SCIEX) was used for quantification.

### Statistical Analysis

The metabolomic raw data were converted to the mzXML format with ProteoWizard software (27) version 3.0 (https://proteowizard.sourceforge.io/) and processed with an in-house program, which was developed using R software version 3.6.3 and based on XCMS software version 3.6.1. In-house MS2 database was applied for metabolite annotation. The resulting dataset including the information of sample name, peak number and normalized peak area was subjected to multivariate analysis using SIMCA 16.0.2 software package (Sartorius Stedim Data Analytics AB). An unsupervised principal component analysis (PCA) with unit variance scaling was performed to visualize the distribution of the samples and assess the stability of the study. A supervised model of orthogonal projections to latent structures-discriminate analysis (OPLS-DA) with unit variance scaling was applied to maximize the distance between groups and find significantly dysregulated metabolites. A 7-fold cross validation was used to evaluate the reliability of our model. A permutation test was proceeded 200 times to estimate the risk of overfitting.

Then the score of variable importance in the projection (VIP) of the first principal component in OPLS-DA model was calculated. The metabolites with VIP > 1, p < 0.05 (student’s t test) and |log_fold change (FC)| (the absolute value of log_FC) > 1 were considered as significantly dysregulated metabolites. Hierarchical clustering analysis was performed to represent the pattern of the dysregulated metabolites among samples, and the volcano plot was constructed to visualize these metabolites. In addition, the MetaboAnalyst database (http://www.metaboanalyst.ca/) were used to reveal the critical disturbed metabolic pathways in MetS(+) HCC. The chord plot and correlation analysis were conducted using R software version 3.6.3.

For targeted metabolite analysis, data were analyzed using GraphPad Prime 5.01 (GraphPad Software) or SPSS 20.0 software (IBM). All data were expressed as mean ± standard deviation (SD). Student’s t test or Mann-Whitney U test was applied to evaluate the differences between two groups, depending on whether the data followed the Gaussian distribution. Receiver operating characteristic (ROC) curves were drawn and the areas under the ROC curves (AUCs) were determined to evaluate the diagnostic performances of the dysregulated metabolites. Youden’s Indices were calculated to determine the cut-off points with optimal sensitivity and specificity. The correlations between the metabolites and clinical indicators were also investigated. The p value < 0.05 was regarded as statistically significant.

## Results

### Serum Metabolic Profiling Identified Significantly Dysregulated Metabolites in MetS(+) HCC

**Figure 1:**
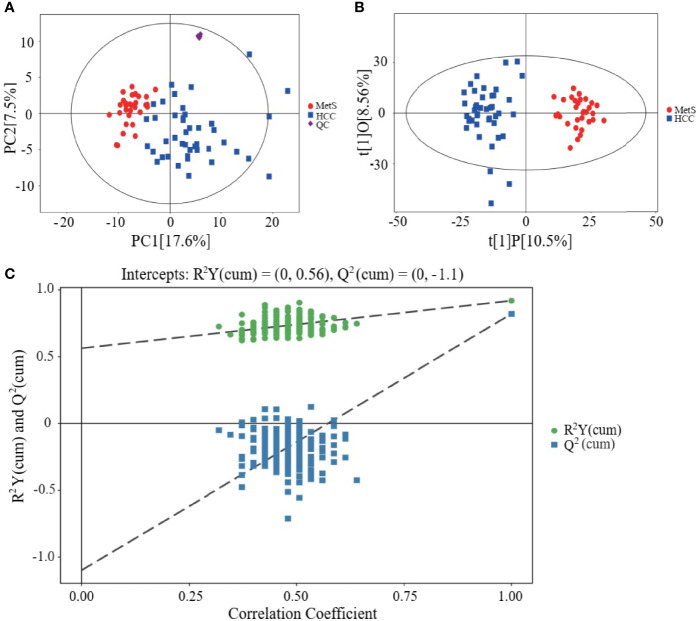
Metabolic profiling of serum samples from patients with MetS and MetS(+) HCC. **(A)** Score plot of principal component analysis (PCA) based on the combinational data of ESI+ and ESI- modes. **(B)** Score plot of orthogonal projections to latent structures-discriminate analysis (OPLS-DA). **(C)** Statistical validation of the OPLS-DA model in 200 random permutation tests.

Metabolic profiling of serum samples from patients with MetS and MetS(+) HCC in the discovery cohort was performed, and pooled QC samples were inserted into batches to evaluate the stability of the analytical method. Representative base peak chromatograms from ESI+ and ESI- mode were displayed in **Supplementary Figure 1**. The QC samples were clustered together in the PCA score plot (**Figure 1A**), indicating that the present analytical method was stable and repeatable. In addition, the reliability of the metabolomic analyses was further confirmed by the high correlation coefficients of the QC samples under the ESI+ and ESI- mode (**Supplementary Figure 2**). Subsequently, the supervised OPLS-DA model was applied to explore the metabolic changes in MetS(+) HCC patients compared to MetS population. As shown in **Figure 1B**, the HCC group was clearly separated from the MetS group in the OPLS-DA score plot, and the cumulative R^2^Y and Q^2^ were 0.92 and 0.82 respectively, representing a high predictive ability of the model. Then the permutation test was conducted 200 times and no overfitting was observed as the cumulative R^2^Y-intercept and Q^2^-intercept were 0.56 and -1.10 respectively (**Figure 1C**).

**Figure 2:**
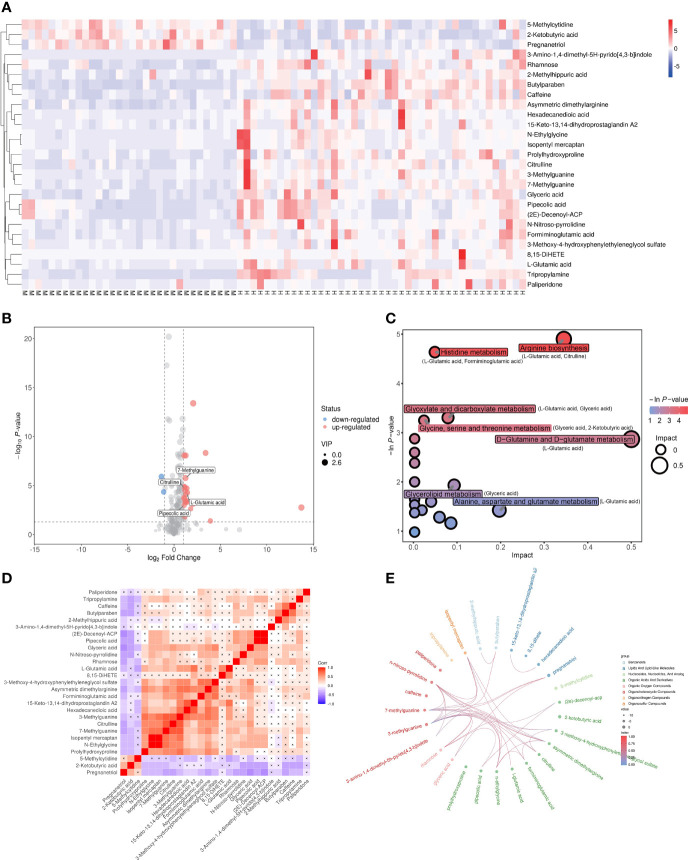
Dysregulated metabolites between MetS and MetS(+) HCC patients identified in metabolic profiling. **(A)** Hierarchical clustering showing the dysregulated metabolites between MetS and MetS(+) HCC patients. Each column represents a sample, and each row represents a metabolite. M represents MetS individual, and H represents MetS(+) HCC patient. ‘Red’ indicates a high level, and ‘blue’ indicates a low level. **(B)** The volcano plot depicts the difference of metabolites between MetS and MetS(+) HCC patients. Red points (up-regulated) and blue points(down-regulated) refer to significant dysregulation according to fold change > 2.0 and p value < 0.05. **(C)** Pathway enrichment analysis of differential metabolites identified in MetS(+) HCC versus MetS group. **(D)** Correlation analysis of differential metabolites identified in MetS(+) HCC versus MetS group. ‘Red’ indicates a positive correlation, and ‘blue’ indicates a negative correlation. **(E)** Chord plot analysis of differential metabolites identified in MetS(+) HCC versus MetS group.

The dysregulated metabolites were selected according to the conditions of VIP > 1, p < 0.05 and |log_FC| > 1, and a total of 27 candidate metabolites including 24 upregulated and 3 downregulated in MetS(+) HCC patients were identified (**Supplementary Table 2**). The result of hierarchical clustering depicted the distinguishable profiling of the dysregulated metabolites between MetS and MetS(+) HCC group (**Figure 2A**), and a volcano plot was constructed for visualizing these metabolites (**Figure 2B**). In addition, pathway analysis was conducted using MetaboAnalyst database, and several metabolic pathways, including arginine biosynthesis, histidine metabolism, glycine, serine and threonine metabolism, and D-glutamine and D-glutamate metabolism, were revealed to be disturbed in MetS(+) HCC compared to MetS patients (**Figure 2C**). Moreover, the chord plot and correlation analysis showed that there were varying levels of correlation among these metabolites (**Figures 2D, E**).

### Validation of Dysregulated Metabolites by Targeted Metabolite Analyses

**Figure 3:**
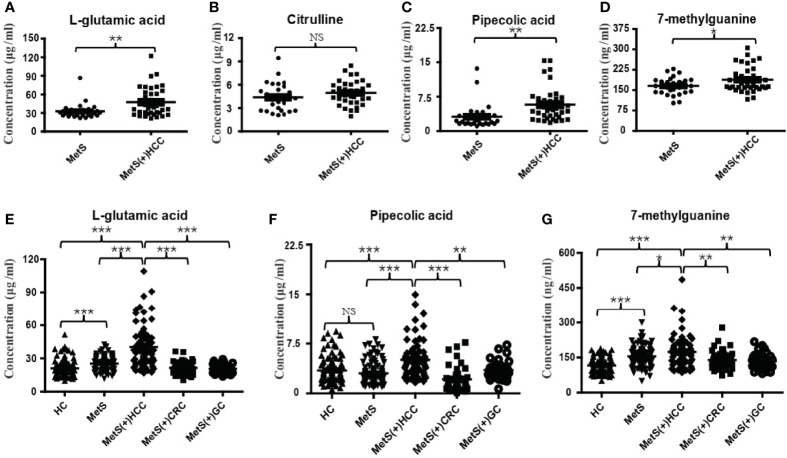
Expression profiles of some dysregulated metabolites in the discovery and validation cohorts. **(A)** The levels of serum L-glu in the discovery cohort. **(B)** The levels of serum Citru in the discovery cohort. **(C)** The levels of serum PA in the discovery cohort. **(D)** The levels of serum 7-mG in the discovery cohort. **(E)** The levels of serum L-glu in the validation cohort. **(F)** The levels of serum PA in the validation cohort. **(G)** The levels of serum 7-mG in the validation cohort. NS, not significant. *p value < 0.05; **p value < 0.01; ***p value < 0.001.

Among these 27 dysregulated metabolites, L-glu, Citru, PA and 7-mG have been reported to be associated with HCC (28–31). To validate the metabolic profiling results, a new method for simultaneous quantification of L-glu, Citru, PA and 7-mG by liquid chromatography tandem mass spectrometry (LC-MS/MS) was developed, and the results of methodology validation suggested the precision and accuracy of the LC-MS/MS method was acceptable (**Supplementary Table 3**). Consistently, the levels of L-glu (**Figure 3A**), PA (**Figure 3C**) and 7-mG (**Figure 3D**) were significantly increased in MetS(+) HCC patients compared to MetS individuals in the discovery cohort. However, there was no significant difference in the levels of Citru between the two groups (**Figure 3B**). In addition, an independent validation cohort was introduced to further confirm the above results. As shown in **Figures 3E–G**, the levels of L-glu, PA and 7-mG were also upregulated significantly in the MetS(+) HCC patients compared to MetS and HC individuals. Interestingly, we found that L-glu and 7-mG were upregulated significantly in MetS compared to HC individuals, while PA showed no significant difference between MetS and HC group, indicating that the levels of L-glu and 7-mG, but not PA, were further affected as a consequence of the MetS in HCC patients. Moreover, as MetS was closely associated with the occurrence of CRC and GC as well as HCC (32, 33), CRC and GC patients were included in this study to assess the specificity of these potential biomarkers for MetS(+) HCC. The results showed that the levels of L-glu, PA and 7-mG in MetS(+) CRC and MetS(+) GC patients were significantly lower than those in MetS(+) HCC patients, suggesting the specificity of these metabolites for the detection of MetS(+) HCC. These results suggested that L-glu, PA and 7-mG were specifically upregulated in MetS(+) HCC.

### Evaluation of the Diagnostic Performances of L-glu, PA, and 7-mG

**Figure 4:**
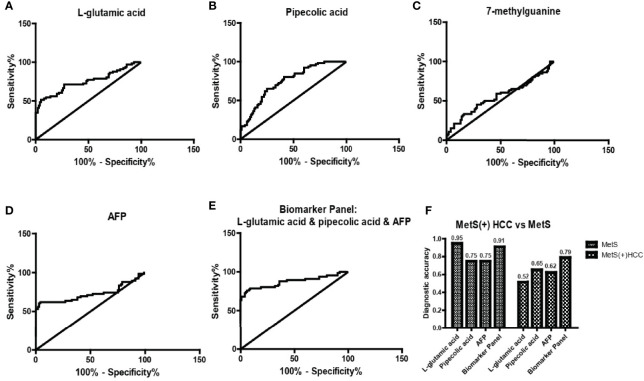
The diagnostic performance of L-glu, PA and 7-mG. **(A)** The ROC curve of L-glu for discriminating MetS(+) HCC from MetS. **(B)** The ROC curve of PA for discriminating MetS(+) HCC from MetS. **(C)** The ROC curve of 7-mG for discriminating MetS(+) HCC from MetS. **(D)** The ROC curve of AFP for discriminating MetS(+) HCC from MetS. **(E)** The ROC curve of the biomarker panel including L-glu, PA and AFP for discriminating MetS(+) HCC from MetS. **(F)** The diagnostic accuracy of L-glu, PA, AFP and the biomarker panel for the diagnosis of MetS and MetS(+) HCC, respectively. ROC, receiver operating characteristic.

Then we evaluated the diagnostic potential of L-glu, PA and 7-mG in MetS(+) HCC using ROC curves. As shown in **Figure 4A** and **Table 2**, L-glu exhibited an AUC of 0.75 in discriminating MetS(+) HCC patients from MetS individuals, and the optimal sensitivity and specificity were 51.52% and 95.00%, respectively. The diagnostic accuracy of L-glu was high (0.95) for MetS patients, but low (0.52) for MetS(+) HCC patients (**Figure 4F**). In addition, PA exhibited an AUC of 0.75 as well, and the optimal sensitivity and specificity were 65.15% and 75.00%, respectively (**Figure 4B** and **Table 2**). The diagnostic accuracy of PA was 0.75 for MetS patients and 0.65 for MetS(+) HCC patients (**Figure 4F**). However, 7-mG showed poor diagnostic performance with an AUC of 0.56 in discriminating MetS(+) HCC patients from MetS individuals (**Figure 4C** and **Table 2**).

**Table 2:** The diagnostic performance of serum metabolites, AFP or their combination for HCC detection in MetS population.

	AUC (95%CI)	Sensitivity (%)	Specificity (%)	p value
L-glu	0.75 (0.67-0.83)	51.52	95.00	<0.0001
PA	0.75 (0.68-0.82)	65.15	75.00	<0.0001
7-mG	0.56 (0.47-0.65)	45.45	72.00	0.2005
AFP	0.73 (0.64-0.82)	62.12	76.00	<0.0001
L-glu & PA & AFP	0.87 (0.81-0.94)	78.79	91.00	<0.0001

As the diagnostic efficacy of AFP was limited (AUC 0.73, sensitivity 62.12% and specificity 76% at the cut-off value of 7.0 ng/ml) (**Figure 4D** and **Table 2**), especially for AFP-negative [AFP(-)] HCC (34), we evaluated the diagnostic potential of L-glu and PA in AFP(-) MetS(+) HCC patients. As shown in **Supplementary Figure 3**, L-glu exhibited an AUC of 0.73 and PA exhibited an AUC of 0.68 in discriminating AFP(-) MetS(+) HCC patients from MetS individuals. Next, we determined whether the combination of L-glu, PA and AFP could improve the accurate diagnosis rate of MetS(+) HCC. Logistic regression based on L-glu, PA and AFP for MetS(+) HCC diagnosis was used to construct a model. This biomarker panel for the detection of MetS(+) HCC was constructed as follows: logit [p = HCC] = 0.0017 × [L-glu] + 0.0021 × [PA] + 0.1216 × [AFP] − 5.6391. As shown in **Figure 4E**, the biomarker panel exhibited better diagnostic performance than AFP alone in differentiating MetS(+) HCC patients from MetS individuals (AUC 0.87, sensitivity 78.79% and specificity 91.00% at the optimal cut-off point) (**Figure 4E** and **Table 2**). More importantly, the biomarker panel showed better diagnostic accuracy than any single biomarker. For MetS and MetS(+) HCC patients, the diagnostic accuracy of the biomarker panel was 0.91 and 0.79, respectively (**Figure 4F**). In general, the diagnostic accuracy of the biomarker panel was similar to that of L-glutamic acid alone (0.91 vs 0.95), but much higher than that of pipecolic acid alone (0.91 vs 0.75) or AFP (0.91 vs 0.75) for MetS patients. Consistently, the diagnostic accuracy of the biomarker panel was much higher than that of L-glutamic acid alone (0.79 vs 0.52), pipecolic acid alone (0.79 vs 0.65) or AFP (0.79 vs 0.62) for MetS(+) HCC patients.

Subsequently, correlation analyses of the score of the biomarker panel with clinical characteristics were performed. As shown in **Table 3**, the biomarker panel was significantly correlated with tumor number, and the higher the score of the biomarker panel was, the more intrahepatic metastases might occur. However, no significant correlation of the score of the biomarker panel with other clinical characteristics of HCC patients was observed, such as tumor size, clinical stage, HBV and HCV status, cirrhosis, alcohol consumption, Child-Pugh classification and the metabolic situations. Together, these results suggested that the biomarker panel including L-glu, PA and AFP had good diagnostic performance for the detection of MetS(+) HCC in MetS population, and it might be associated with multiple intrahepatic metastases of HCC.

**Table 3:** Correlation of the score of the biomarker panel with clinical variables in MetS(+) HCC patients in the validation cohort.

Variables	N	Biomarker Panel Score		P value
		Low (n = 33)	High (n = 33)	
**Age**				0.45
≤60 y	40	18	22	
>60 y	26	15	11	
**Gender**				0.22
Male	53	29	24	
Female	13	4	9	
**FG (mmol/L)**				0.42
		7.23 ± 2.48	6.65 ± 2.91	
**TG (mmol/L)**				0.20
		1.33 ± 0.57	1.56 ± 0.81	
**HDL-C (mmol/L)**				0.77
		0.95 ± 0.23	0.93 ± 0.26	
**Central obesity**				0.80
Yes	40	21	19	
No	26	12	14	
**Hypertension**				0.79
Yes	44	21	23	
No	22	12	10	
**HBV**				0.61
Positive	43	20	23	
Negative	23	13	10	
**HCV**				1.00
Positive	1	1	0	
Negative	65	32	33	
**Cirrhosis**				1.00
Yes	38	19	19	
No	28	14	14	
**Alcohol Consumption**				0.30
Yes	10	3	7	
No	56	30	26	
**Child-Pugh Classification**				0.62
A	35	19	16	
B-C	31	14	17	
**Tumor Size**				0.13
>5 cm	27	10	17	
≤5 cm	39	23	16	
**Tumor Number**				0.01^*^
=1		29	19	
>1		4	14	
**Clinical Stage**				0.08
I-II	26	17	9	
III-IV	40	16	24	
**Differentiation**				0.43
High-Moderate	44	24	20	
Low	22	9	13	
**Vascular invasion**				0.32
Yes	35	15	20	
No	31	18	13	

^*^P value < 0.05.

### Diagnostic Performance of the Biomarker Panel in Small and Early-Stage MetS(+) HCC

**Figure 5:**
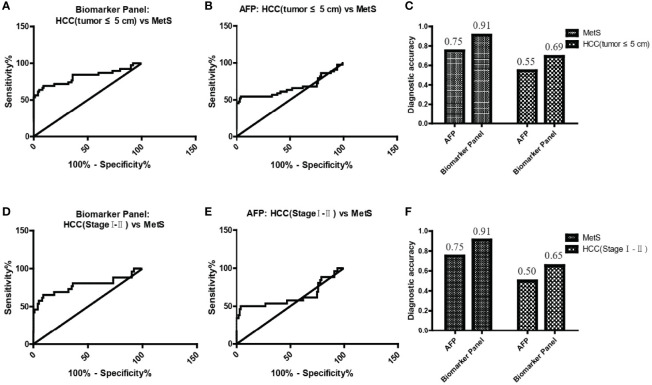
The role of the biomarker panel in the diagnosis of small and early-stage HCC patients. **(A)** The ROC curve of the biomarker panel for discriminating small (tumor ≤ 5 cm) MetS(+) HCC patients from MetS individuals. **(B)** The ROC curve of AFP for discriminating small (tumor ≤ 5 cm) MetS(+) HCC patients from MetS individuals. **(C)** The diagnostic accuracy of AFP and the biomarker panel for the diagnosis of MetS and small (tumor ≤ 5 cm) MetS(+) HCC, respectively. **(D)** The ROC curve of the biomarker panel for discriminating early-stage (stage I-II) MetS(+) HCC patients from MetS individuals. **(E)** The ROC curve of AFP for discriminating early-stage (stage I-II) MetS(+) HCC patients from MetS individuals. **(F)** The diagnostic accuracy of AFP and the biomarker panel for the diagnosis of MetS and early-stage (stage I-II) MetS(+) HCC, respectively. ROC, receiver operating characteristic.

As small and early-stage HCC patients are often difficult to detect, we evaluated the diagnostic value of the biomarker panel in these tumors. As shown in **Figure 5A** and **Table 4**, the biomarker panel exhibited an AUC of 0.82 in discriminating small MetS(+) HCC from MetS, and the optimal sensitivity and specificity were 69.23% and 91.00%, respectively. However, AFP showed much poorer diagnostic performance than the biomarker panel in discriminating small MetS(+) HCC from MetS, and the AUC, sensitivity and specificity were 0.67, 54.55% and 76.00%, respectively (**Figure 5B** and **Table 4**). More importantly, the diagnostic accuracy of the biomarker panel was 0.91 for MetS patients and 0.69 for small MetS(+) HCC patients, which was much higher than that of AFP(**Figure 5C**).

**Table 4:** The diagnostic performance of the biomarker panel for the detection of small and early-stage HCC in MetS population.

Groups	AUC (95%CI)	Sensitivity (%)	Specificity (%)	p value
**AFP**				
HCC (tumor ≤ 5 cm) vs MetS	0.67 (0.55-0.78)	54.55	76.00	0.001319
HCC (Stage I-II) vs MetS	0.63 (0.48-0.78)	50.00	76.00	0.03986
**Biomarker Panel**				
HCC (tumor ≤ 5 cm) vs MetS	0.82 (0.72-0.91)	69.23	91.00	<0.0001
HCC (Stage I-II) vs MetS	0.78 (0.65-0.91)	65.38	91.00	<0.0001

In addition, the biomarker panel exhibited an AUC of 0.78 in discriminating early-stage (stage I-II) MetS(+) HCC from MetS, and the optimal sensitivity and specificity were 65.38% and 91.00%, respectively (**Figure 5D** and **Table 4**). Consistently, AFP showed much poorer diagnostic performance than the biomarker panel in discriminating early-stage MetS(+) HCC from MetS, and the AUC, sensitivity and specificity were 0.63, 50.00% and 76.00%, respectively (**Figure 5E** and **Table 4**). The diagnostic accuracy of the biomarker panel was 0.91 for MetS patients and 0.65 for early-stage MetS(+) HCC patients, which were much higher than that of AFP(**Figure 5F**), indicating that the results with early-stage MetS(+) HCC were similar to the results with small MetS(+) HCC. Collectively, these findings indicated the importance of the biomarker panel in the diagnosis of small and early-stage MetS(+) HCC.

### The Specificity of the Biomarker Panel for MetS(+) HCC

**Figure 6:**
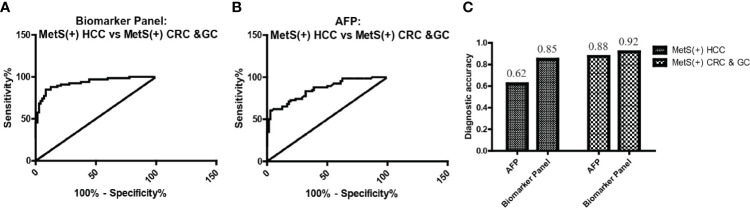
The specificity of the biomarker panel in the diagnosis of MetS(+) HCC patients. **(A)** The ROC curve of the biomarker panel for discriminating MetS(+) HCC patients from MetS(+) CRC & GC patients. **(B)** The ROC curve of AFP for discriminating MetS(+) HCC patients from MetS(+) CRC & GC patients. **(C)** The diagnostic accuracy of AFP and the biomarker panel for the diagnosis of MetS(+) HCC and MetS(+) CRC & GC, respectively. ROC, receiver operating characteristic.

As L-glu and PA were specifically upregulated in MetS(+) HCC, but not MetS(+) CRC and MetS(+) GC, we then evaluated the specificity of biomarker panel for MetS(+) HCC. As shown in **Figure 6A** and **Table 5**, the biomarker panel exhibited an AUC of 0.93 in discriminating MetS(+) HCC from MetS(+) CRC & GC, and the optimal sensitivity and specificity were 84.85% and 91.67%, respectively. It showed better diagnostic performance than AFP alone, the AUC, sensitivity and specificity of which were 0.85, 62.12% and 87.50%, respectively (**Figure 6B** and **Table 5**). The diagnostic accuracy of the biomarker panel was 0.85 for MetS(+) HCC patients and 0.92 for MetS(+) CRC & GC patients, which were higher than that of AFP as well (**Figure 6C**). These data clearly suggested the high specificity of the biomarker panel for the diagnosis of MetS(+) HCC.

**Table 5:** The diagnostic performance of the biomarker panel for discriminating MetS(+) HCC from MetS(+) CRC & GC.

	AUC (95%CI)	Sensitivity (%)	Specificity (%)	p value
**AFP**	0.85(0.79-0.92)	62.12	87.50	<0.0001
**Biomarker Panel**	0.93(0.89-0.97)	84.85	91.67	<0.0001

## Discussion

In this study, serum L-glu and PA were found to be upregulated in MetS(+) HCC patients compared to MetS(+) individuals. Dysregulated L-glu and PA showed adequate diagnostic performance in differentiating MetS(+) HCC from MetS(+) individuals, and the combination of L-glu, PA and AFP exhibited much better diagnostic performance than AFP alone. The biomarker panel also offered high diagnostic accuracy in the detection of small and early-stage MetS(+) HCC. The specificity of the biomarker panel was high for MetS(+) HCC as it distinguished patients with MetS(+) HCC from patients with MetS(+) CRC & GC accurately.

It is essential to explore potential biomarkers for HCC surveillance in MetS population because of the high prevalence of MetS worldwide and the close association of MetS with HCC (4, 35). Currently, there is no effective diagnostic marker for MetS(+) HCC in clinic, and the screening of small and early-stage tumors remains a challenge. In the present study, we found that L-glu, PA and 7-mG were upregulated in MetS(+) HCC. However, they were not uniformly higher in MetS(+) HCC patients compared to MetS individuals, and no single marker would be sufficient to identify patients with tumors. Therefore, we constructed a biomarker panel including L-glu, PA and AFP, which could effectively discriminate patients with MetS(+) HCC from the high-risk MetS population. In addition, it was able to detect small and early-stage MetS(+) HCC with high sensitivity and specificity. This study highlights the early diagnostic potential of this biomarker panel. It will help avoid delays in treatment and progression of the disease, and exert an important impact on prognosis improvement of MetS(+) HCC patients.

A growing number of studies have described the important role of serum metabolites in the diagnosis of HCC. For example, the specific changes in serum concentrations of several amino acids and lipids, including glycine, aspartic acid, sphingomyelin (42:3), and sphingomyelin (43:2), are useful for the early diagnosis of HCC (36). In addition, it has been reported by another group that the serum metabolite panel including phenylalanyl-tryptophan and glycocholate, as well as the combination of betaine and propionylcarnitine, conferred good diagnostic potential to discriminate HCC from chronic hepatitis and cirrhosis (18, 37). Moreover, the combination of retinol and retinal, and the serum metabolite panel including chenodeoxycholic acid, lysophosphatidylcholine (20:5), succinyladenosine and uridine were identified as potential diagnostic tools for HCC as well (20, 38). The results of these metabolomic studies were inconsistent, which might be due to the unreliability of untargeted metabolomics and the differences in sample selection. In this study, we verified the results of untargeted metabolomics with targeted LC-MS/MS analyses to ensure the analytical accuracy. In addition, as the aim of this study was to explore novel diagnostic markers for MetS(+) HCC, all included HCC patients had MetS. In this study, we found that serum L-glu and PA was dysregulated in MetS(+) HCC compared to MetS group, and might be specific for the diagnosis of MetS(+) HCC individuals.

The molecular mechanism by which MetS induced the development of HCC remains intriguing. It is well established that insulin resistance exerts a critical role in the pathogenesis of HCC by increasing insulin growth factor-1 (IGF-1), which has important proliferative, antiapoptotic and angiogenesis effects (39). In addition, obesity promotes liver inflammation and tumorigenesis by enhancing the expression of interleukin-6 (IL-6) and tumor necrosis factor (TNF), which further activates several pro-oncogenic pathways (40, 41). Previous studies have also demonstrated that small molecule metabolites were important for MetS(+) HCC tumorigenesis. For example, MetS played an important role in HBV-associated HCC tumorigenesis, and the dysregulation of lipid metabolic genes and lipid (triglycerides, cholesterol, and fatty acids) profiles might promote the occurrence of HCC in chronic hepatitis B patients (42). In addition, 8-hydroxydeoxyguanosine, L-arginine and glucose metabolites were found to be upregulated in MetS and non-alcoholic steatohepatitis-associated hepatocarcinogenesis, and might take part in various tumor-associated processes, including the activation of oxidative stress resistance, mTOR pathways and cell proliferation (43). However, these studies were mainly conducted in mice model, and the dysregulation in MetS(+) HCC compared to MetS patients is still largely unclear at present. In this study, we compared the serum metabolite profiles in MetS patients and MetS(+) HCC patients, and found that some metabolites and pathways were disturbed significantly in MetS(+) HCC patients (**Figure 2**), indicating that they might influence the HCC tumorigenesis in MetS population.

Amino acids and their derivatives are usually aberrantly regulated in cancer, and play a key role in the diagnosis of various cancers (44, 45). However, their dysregulation in HCC is still controversial. For example, L-glu participated in several metabolic pathways, including arginine biosynthesis, histidine metabolism and D-glutamine and D-glutamate metabolism (**Figure 2C**), and several studies reported that L-glu was significantly increased in HCC compared with cirrhosis (46, 47), but some other studies indicated that it was downregulated (20) or had no significant change (18, 48) in HCC. PA, which is a metabolite of lysine degradation, was also found significantly differed between HCC and the other two cohorts of health and cirrhosis group (49). However, it showed no significant difference in other studies (18, 20). The inconsistency of these studies might be due to different included HCC patients. In this study, we recruited MetS(+) HCC patients specifically to explore the metabolite features in this type of HCC. Both L-glu and PA were upregulated in MetS(+) HCC compared to MetS individuals, indicating the alterations in particular metabolic pathways, such as arginine biosynthesis, histidine metabolism, D-glutamine and D-glutamate metabolism and lysine degradation. Our results further support the potential of amino acids and their derivatives as cancer biomarkers.

In conclusion, the present study is the first to demonstrate that serum L-glu and PA are upregulated in MetS(+) HCC patients, and biomarker panel including L-glu, PA and AFP exhibits good diagnostic performance for discriminating MetS(+) HCC from MetS patients. In addition, the biomarker panel is specific for MetS(+) HCC, but not MetS(+) CRC or MetS(+) GC. Therefore, this biomarker panel has great promise for clinical application in MetS(+) HCC diagnosis. In the future, large-scale, multicenter prospective studies will be needed to further confirm our findings.

## Data Availability Statement

The original contributions presented in the study are included in the article/**Supplementary Material**. Further inquiries can be directed to the corresponding author.

## Ethics Statement

The studies involving human participants were reviewed and approved by The Ethics Committee of Peking University People’s Hospital. The patients/participants provided their written informed consent to participate in this study.

## Author Contributions

L-LC conceived and designed the experiments. YH, YW, LP, and LQ collected the clinical samples. L-LC, YH, BL, and JC performed the experiments. ZY, MJ, and L-LC analyzed the data. L-LC and HW wrote the manuscript. ZY, MJ, and HW revised the manuscript. All authors contributed to the article and approved the submitted version.

## Funding

This work was supported by the National Natural Science Foundation of China grant 81702788.

## Conflict of Interest

JC was employed by SCIEX Analytical Instrument Trading Co., Shanghai, China.

The remaining authors declare that the research was conducted in the absence of any commecial or financial relationships that could be construed as a potential conflict of interest.

## Publisher’s Note

All claims expressed in this article are solely those of the authors and do not necessarily represent those of their affiliated organizations, or those of the publisher, the editors and the reviewers. Any product that may be evaluated in this article, or claim that may be made by its manufacturer, is not guaranteed or endorsed by the publisher.

## Supplementary Material

Supplementary Figure 1 Representative base peak chromatograms under the ESI+ **(A)** and ESI- **(B)** mode.

Supplementary Figure 2 The correlation coefficients of the QC samples under the ESI+ **(A)** and ESI- **(B)** mode.

Supplementary Figure 3 The diagnostic performance of L-glu **(A)** and PA **(B)** for discriminating AFP(-) MetS(+) HCC from MetS.
