# Supplementary figures and images for: Metabolic Profiling Identified a Novel Biomarker Panel for Metabolic Syndrome-Positive Hepatocellular Cancer
Source: Front Endocrinol (Lausanne). 2022 Jan 26;12:816748. doi: 10.3389/fendo.2021.816748 (PMC8826723; doi:10.3389/fendo.2021.816748)

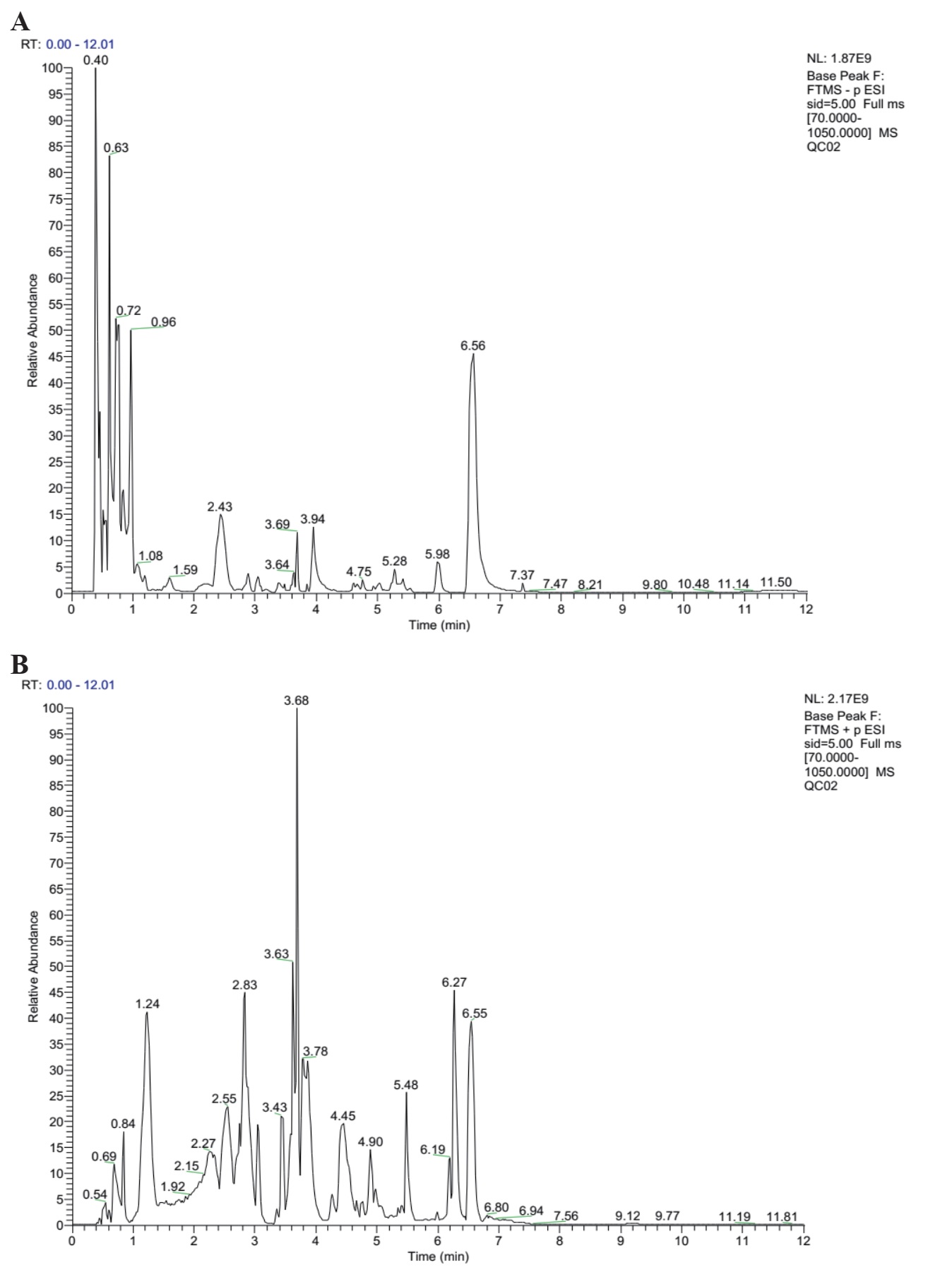

Supplement: Supplementary Figure 1 — Representative base peak chromatograms under the ESI+ (A) and ESI- (B) mode. [file Image_1.jpeg]

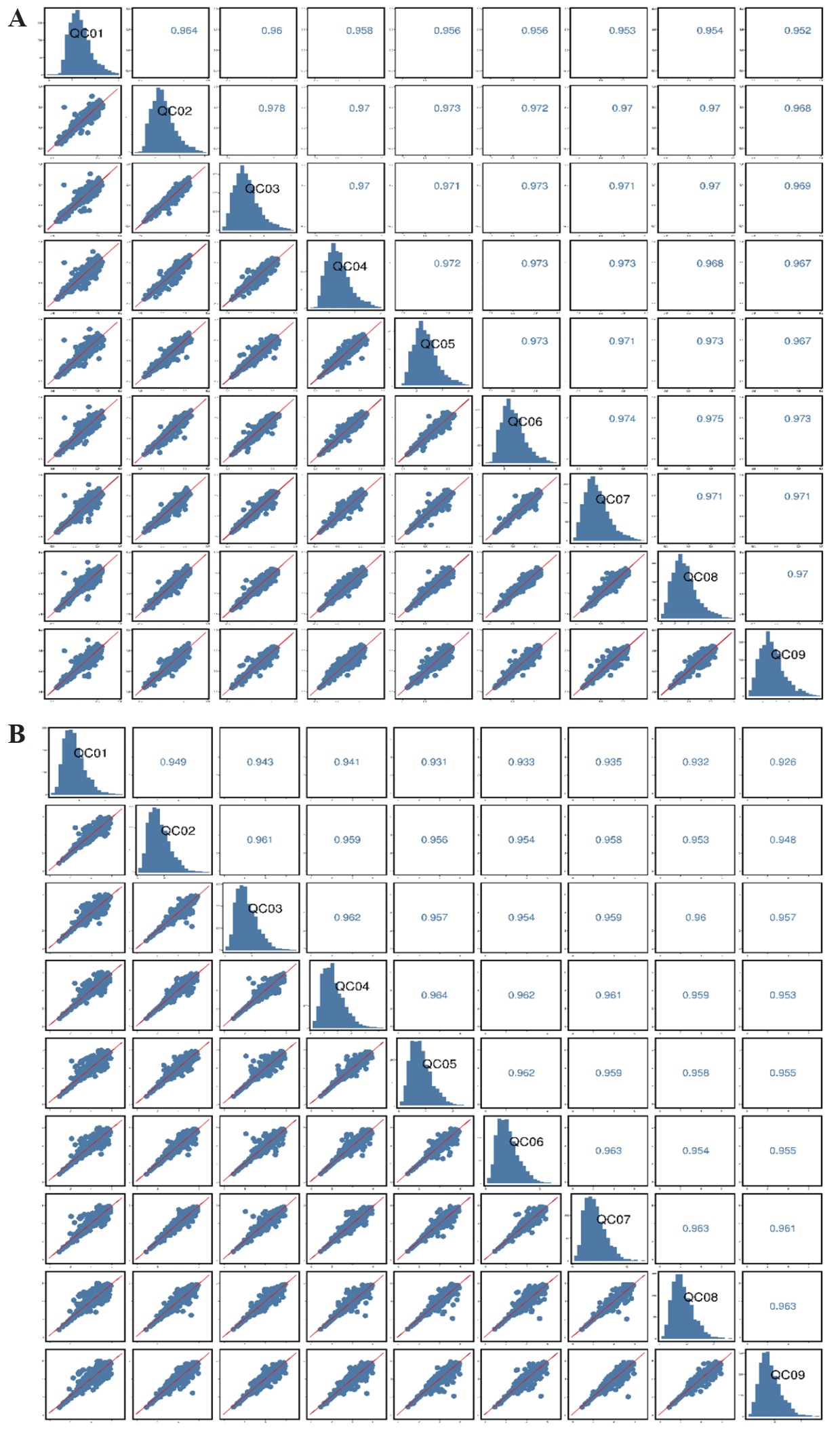

Supplement: Supplementary Figure 2 — The correlation coefficients of the QC samples under the ESI+ (A) and ESI- (B) mode. [file Image_2.jpeg]

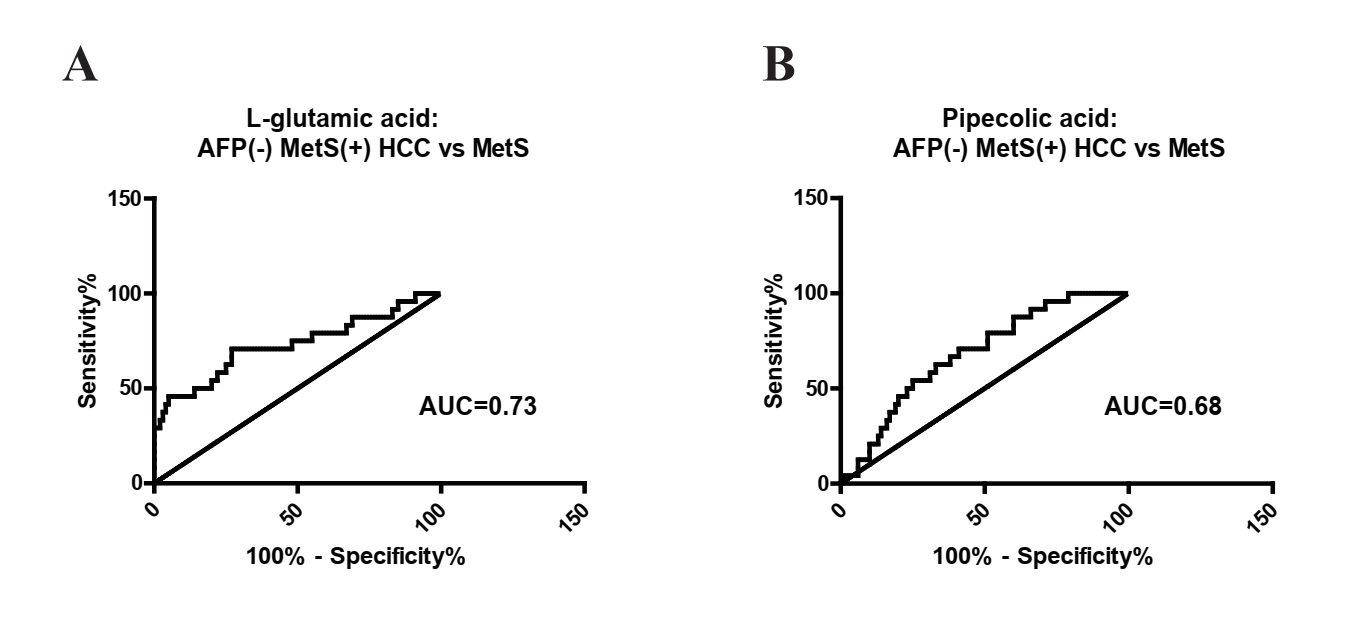

Supplement: Supplementary Figure 3 — The diagnostic performance of L-glu (A) and PA (B) for discriminating AFP(-) MetS(+) HCC from MetS. [file Image_3.jpg]
