# Supplementary material for: Metabolic Profiling Identified a Novel Biomarker Panel for Metabolic Syndrome-Positive Hepatocellular Cancer
Source: Front Endocrinol (Lausanne). 2022 Jan 26;12:816748. doi: 10.3389/fendo.2021.816748 (PMC8826723; doi:10.3389/fendo.2021.816748)
Supplement: Supplementary file 4 [file Table_1.docx]

**Supplementary Table 1. MRM settings for L-glutamic acid, citrulline, pipecolic acid and 7-methylguanine**

| Q1 Mass (Da) | Q3 Mass (Da) | Dwell Time (msec) | ID | DP (volts) | EP (volts) | CE (volts) | CXP (volts) |
| --- | --- | --- | --- | --- | --- | --- | --- |
| 148 | 130.2 | 25 | L-glutamic acid-1 | 47 | 10 | 14 | 15 |
| 148 | 84.1 | 25 | L-glutamic acid-2 | 47 | 10 | 22 | 15 |
| 153 | 87.9 | 25 | L-glutamic acid-d5-1 | 40 | 10 | 20 | 8 |
| 153 | 135.1 | 25 | L-glutamic acid-d5-2 | 40 | 10 | 14 | 8 |
| 176 | 159.2 | 25 | citrulline-1 | 52 | 10 | 15 | 15 |
| 176 | 113.3 | 25 | citrulline-2 | 52 | 10 | 23 | 15 |
| 166 | 149.1 | 25 | 7-methylguanine-1 | 79 | 10 | 28 | 15 |
| 166 | 124.2 | 25 | 7-methylguanine-2 | 79 | 10 | 29 | 15 |
| 130.1 | 84.2 | 25 | pipecolic acid-1 | 69 | 10 | 25 | 15 |
| 130.1 | 56.2 | 25 | pipecolic acid-2 | 69 | 10 | 20 | 15 |
