# Supplementary material for: Metabolic Profiling Identified a Novel Biomarker Panel for Metabolic Syndrome-Positive Hepatocellular Cancer
Source: Front Endocrinol (Lausanne). 2022 Jan 26;12:816748. doi: 10.3389/fendo.2021.816748 (PMC8826723; doi:10.3389/fendo.2021.816748)
Supplement: Supplementary file 5 [file Table_2.docx]

**Supplementary Table 2. List of differential metabolites identified in the metabolomic analyses**

| **Metabolite** | **MS2 score** | **SuperClass** | **VIP** | **P Value** | **FC** | **LOG_FC** |
| --- | --- | --- | --- | --- | --- | --- |
| 2-Ketobutyric acid | 0.996978923 | Organic acids and derivatives | 1.952835 | 4.14E-06 | 0.484531 | -1.045338 |
| 3-Methylguanine | 0.975778077 | Organoheterocyclic compounds | 1.803406 | 0.000166 | 2.111139 | 1.07802 |
| Asymmetric dimethylarginine | 0.866930077 | Organic acids and derivatives | 2.143563 | 8.69E-09 | 2.12623 | 1.088297 |
| Pipecolic acid | 0.998462154 | Organic acids and derivatives | 1.461918 | 0.001002 | 2.130279 | 1.091043 |
| Caffeine | 0.602245077 | Organoheterocyclic compounds | 1.558587 | 3.34E-07 | 2.132519 | 1.092558 |
| Prolylhydroxyproline | 0.771868692 | Organic acids and derivatives | 1.134787 | 1.18E-05 | 2.145964 | 1.101626 |
| 2-Methylhippuric acid | 0.691569 | Benzenoids | 1.733472 | 0.000218 | 2.149618 | 1.104081 |
| 5-Methylcytidine | 0.846486692 | Nucleosides, nucleotides, and analogues | 1.647132 | 4.44E-05 | 0.460535 | -1.118618 |
| Citrulline | 0.988747077 | Organic acids and derivatives | 1.906391 | 1.56E-05 | 2.246454 | 1.16765 |
| Paliperidone | 0.674934 | Organoheterocyclic compounds | 1.192887 | 0.015528 | 2.260076 | 1.176372 |
| N-Nitroso-pyrrolidine | 0.984642769 | Organoheterocyclic compounds | 2.205719 | 2.96E-05 | 2.266114 | 1.180219 |
| 3-Methoxy-4-hydroxyphenylethyleneglycol sulfate | 0.693052615 | Organic acids and derivatives | 1.547783 | 0.000475 | 2.266546 | 1.180496 |
| 7-Methylguanine | 0.818804846 | Organoheterocyclic compounds | 2.242184 | 1.72E-06 | 2.349536 | 1.232374 |
| Glyceric acid | 0.937822385 | Organic oxygen compounds | 2.12207 | 8.71E-09 | 2.395152 | 1.260119 |
| L-Glutamic acid | 0.848529462 | Organic acids and derivatives | 1.583312 | 7.92E-05 | 2.440233 | 1.28702 |
| N-Ethylglycine | 0.984523308 | Organic acids and derivatives | 1.928598 | 0.00041 | 2.518473 | 1.33255 |
| Pregnanetriol | 0.762209615 | Lipids and lipid-like molecules | 1.941073 | 1.24E-06 | 0.390398 | -1.356981 |
| (2E)-Decenoyl-ACP | 0.862094231 | Organic acids and derivatives | 1.566082 | 0.000525 | 2.573393 | 1.363671 |
| Rhamnose | 0.784854615 | Organic oxygen compounds | 1.722937 | 1.91E-05 | 2.58759 | 1.37161 |
| Formiminoglutamic acid | 0.854131692 | Organic acids and derivatives | 1.512142 | 5.15E-05 | 2.72372 | 1.445579 |
| 15-Keto-13,14-dihydroprostaglandin A2 | 0.817320231 | Lipids and lipid-like molecules | 1.333543 | 0.000361 | 2.773425 | 1.471668 |
| Hexadecanedioic acid | 0.900703769 | Lipids and lipid-like molecules | 1.379094 | 0.002281 | 3.466805 | 1.793608 |
| Isopentyl mercaptan | 0.854245692 | Organosulfur compounds | 2.074924 | 0.000733 | 4.156639 | 2.055419 |
| Butylparaben | 0.673308923 | Benzenoids | 2.63314 | 4.06E-14 | 4.190851 | 2.067245 |
| Tripropylamine | 0.972441 | Organonitrogen compounds | 2.138421 | 4.77E-09 | 10.55053 | 3.399241 |
| 8,15-DiHETE | 0.976538385 | Lipids and lipid-like molecules | 1.392368 | 0.040047 | 15.07114 | 3.913717 |
| 3-Amino-1,4-dimethyl-5H-pyrido[4,3-b]indole | 0.706158231 | Organoheterocyclic compounds | 2.485979 | 0.001709 | 1.35E+04 | 13.71926 |

Abbreviations: RT, Retention Time; FC, Fold Change.
