# Supplementary material for: Metabolic Profiling Identified a Novel Biomarker Panel for Metabolic Syndrome-Positive Hepatocellular Cancer
Source: Front Endocrinol (Lausanne). 2022 Jan 26;12:816748. doi: 10.3389/fendo.2021.816748 (PMC8826723; doi:10.3389/fendo.2021.816748)
Supplement: Supplementary file 6 [file Table_3.docx]

**Supplementary Table 3. The results of methodology validation for the quantification of**

**serum L-glutamic acid, citrulline, pipecolic acid and 7-methylguanine**

|  | **Precisions** | | | | **Linear Range**  **(μg/mL)** | **Regression**  **Coefficient (R^2^)** | **Recoveries** | |
| --- | --- | --- | --- | --- | --- | --- | --- | --- |
|  | intra-day | | inter-day | |  |  |  |  |
|  | Low | High | Low | High |  |  | Low | High |
| L-glutamic acid | 6.56% | 2.99% | 9.37% | 7.96% | 4.69-150 | 0.999 | 99.21% | 99.45% |
| Citrulline | 8.21% | 3.47% | 9.19% | 6.97% | 1.17-75 | 0.99 | 92.54% | 93.24% |
| Pipecolic acid | 8.90% | 4.57% | 14.89% | 10.82% | 1.17-75 | 0.99 | 107.94% | 103.09% |
| 7-methylguanine | 5.58% | 3.10% | 8.85% | 8.21% | 0.047-1.5 | 0.999 | 91.27% | 109.09% |
